# Amontons-Coulomb-like slip dynamics in acousto-microfluidics

**DOI:** 10.1038/s41467-022-28823-6

**Published:** 2022-03-22

**Authors:** Aurore Quelennec, Jason J. Gorman, Darwin R. Reyes

**Affiliations:** National Institute of Standards and Technology, Gaithersburg, MD 20899 USA

**Keywords:** Acoustics, Fluid dynamics

## Abstract

Acousto-microfluidics uses acoustic waves to manipulate and sense particles and fluids, and its integration into biomedical technologies has grown substantially in recent years. Fluid manipulation and measurement with surface acoustic waves rely on the efficient transmission of acoustic energy from the device to the fluid. Acoustic transmission into the fluid can be reduced significantly by slip at the fluid-solid interface, but, up until now, this phenomenon has been widely neglected during the design of acousto-microfluidic devices. Here our interpretation supports that the slip dynamics at the liquid-solid interface in acousto-microfluidics are highly analogous to the Amontons-Coulomb laws for dry friction between solids. In particular, there is a relationship between the local fluid pressure and shear stress, where we show that pressure-shear stress conditions can be divided into slip and no-slip regions, similar to the cone of friction found in dry friction. This improved understanding of slip will enable more reliable and predictable acousto-microfluidic technologies, thus expanding their use in new applications in biology and medicine.

## Abstract (web summary)

Acoustic waves can be used to manipulate particles and fluids in biomedical applications. The authors show that slip at the fluid-solid interface, characterized by a lower acoustic transmission into the fluid, is similar to Amontons-Coulomb friction, as found between solids.

## Introduction

Acousto-microfluidic actuators^1–5^ and sensors^6–8^ are emerging tools that will enable fundamental research in biosciences and be used in biomedical diagnostics and therapeutics. For example, acoustic resonators, including quartz crystal microbalances (QCM), are used to measure analyte concentrations in fluids by tracking changes in mechanical resonances^6–8^. More recently, acoustic waves emitted from a resonant device located within a microchannel have been used to efficiently trap, separate, and sort particles in fluids^1–5^. In general, acousto-microfluidic devices leverage the absorption of acoustic energy by the fluid to manipulate the fluid, as well as particles within it, or to detect the properties of the fluid in real-time. The acoustic waves are typically generated with piezoelectric actuators embedded in the substrate that supports the microfluidic network. However, the acousto-fluidic interactions used in these applications are complicated by the presence of slip at the fluid–solid interface, which reduces the absorption of acoustic energy and can dramatically change the behavior of acousto-microfluidic devices.

Slip at the fluid–solid interface without acousto-fluidic interactions has been studied extensively^9–12^, including the role of shear stress^13–15^, wall roughness^16,17^, and surface tension^17,18^. However, the introduction of acoustic waves at the fluid–solid interface dramatically changes the slip dynamics^19–26^ and alters the function of devices^19,26^. Specifically, the slip from acousto-fluidic friction generates lower-than-expected damping, or attenuation, of the acoustic waves^20,21,27,28^. The slip coefficient is defined as the difference in damping with and without slip^20^ and is proportional to the slip length, which is a more physically intuitive parameter for quantifying the slip at the fluid–solid interface^22^. These parameters have been used to characterize slip in several acousto-fluidic systems, including an electroacoustic delay line for studying slip in non-Newtonian liquids^20,21^ and QCMs, where in one example, polymer pillars were fabricated on top of a QCM and the slip between droplets and the pillar array was studied^23^. Previous work has focused on devices without flow and has not looked at fluids confined by a microfluidic channel, except for one study with a suspended QCM sensor^25^ where slip was not observed. In addition, the effects of viscosity, density, pressure, shear stress, and device geometry on slip have largely gone unexplored in acousto-microfluidics, which is limiting the design of efficient, reliable, and predictable devices.

Here we present, for the first time, a set of experiments that show the slip dynamics in acousto-microfluidics under a wide range of operating conditions. We have integrated surface acoustic wave sensors into a microfluidic chip that is used to measure the slip coefficient due to acousto-fluidic friction, where the surface acoustic waves travel at the interface between the vibrating substrate and the fluid. We have investigated two cases, with and without fluid flow in the microchannel, allowing us to observe the complex slip dynamics resulting from acousto-fluidic interactions, which are not well understood^11,12^. In addition, the local acousto-fluidic pressure, shear stress, and sensor contact area with the fluid have been varied. From these detailed experiments, it was found that the slip dynamics in acousto-microfluidics are highly analogous to the Amontons-Coulomb laws for dry friction between solids^29,30^. The Amontons-Coulomb friction laws explain that slip between two solids is the result of a coupling effect between the normal and tangential forces at the contact point, with a coupling coefficient that depends on material properties and surface roughness. Although the relationship between local fluid pressure and shear stress is analogous to the normal and tangential forces, respectively, in the Amontons-Coulomb laws, the two factors have never been studied together in microfluidics until now. Our results show a cone of friction that divides the regions of slip and no-slip, which are defined by a relationship between fluid pressure and shear stress. These observations support the use of a cone of friction representation of the slip dynamics that can be used to develop acousto-microfluidic devices that either avoid slip to maximize the acousto-fluidic interaction, as needed for particle trapping and sorting, or leverage slip as a sensing modality (e.g., dynamic viscometer).

## Results

### Acousto-microfluidic sensors and experimental details

**Fig. 1:**
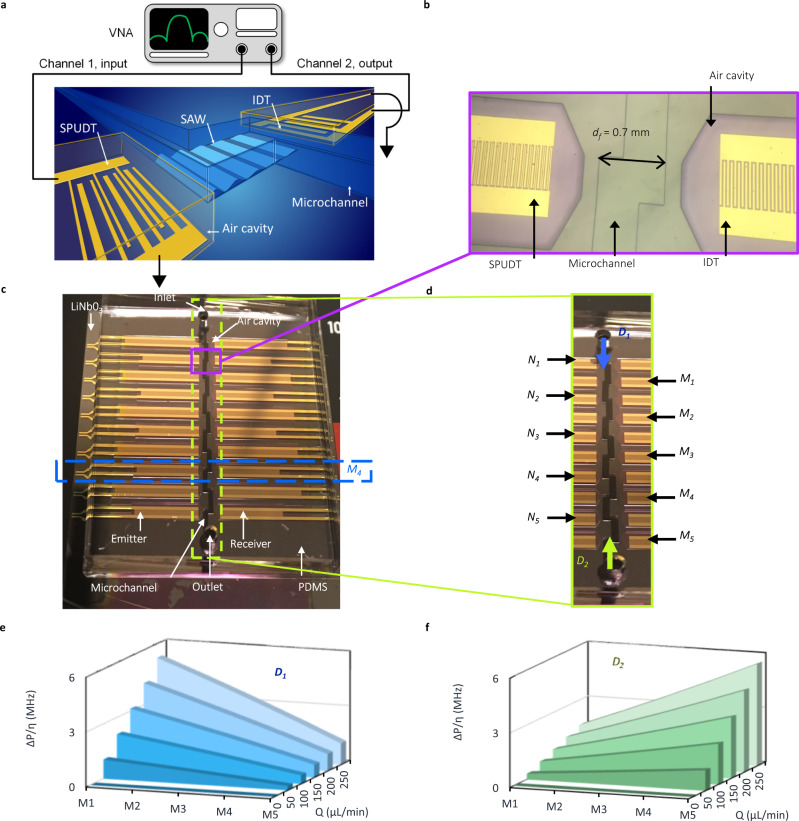
Acousto-microfluidic sensors used to study fluid–solid slip. **a** Diagram of the sensor and associated instrumentation. The sensor is composed of an emitter and a receiver spaced by a delay line. As the surface acoustic wave (SAW) travels a distance *d*_*f*_ in contact with a fluid flow at a flow rate *Q*, its amplitude is damped. The flow at the delay line is parallel to the SAW propagation direction. The emitter and receiver are placed in air cavities to reduce damping from the PDMS. The emitter is a single-phase uni-directional transducer (SPUDT) and the receiver is a standard interdigitated transducer (IDT). Both have the same finger pitch, *λ*, and aperture, *A*. **b** Optical micrograph of one sensor (*M*_1_). The air cavities around the emitter and the receiver, and the microchannel filled with water are visible. **c** Optical image of an assembled sensor array located on one microfluidic channel. Fluid is confined between a PDMS microfluidic channel and the lithium niobate (LiNbO_3_) substrate. **d** Sensor array. There are ten sensors *M*_1_ to *M*_5_ and *N*_1_ to *N*_5_ with different *d*_*f*_ and *λ*. The fluid flows from *M*_1_ to *M*_5_ in direction *D*_1_, and from *M*_5_ to *M*_1_ in direction *D*_*2*_. **e** Calculated local viscous pressure, Δ*P/η*, along *D*_1_. Δ*P* is the pressure drop between the sensor and the atmospheric pressure at the end of the output tubing. Δ*P/η* for different flow rates *Q*, in μL/min, is represented for *M*_1_ to *M*_5_, and for the flow along the direction *D*_1_. **f** Δ*P/η* along *D*_2_. Δ*P/η* for different flow rates *Q*, in μL/min, is represented for *M*_1_ to *M*_5_ for the flow along the direction *D*_2_.

The sensors have a surface acoustic wave delay line configuration (Fig. 1a) with two electroacoustic interdigitated transducers (IDT): one emitter and one receiver, spaced by a distance *d*_*f*_, as shown in Fig. 1b. The fluid flows over the acoustic delay line contained within a polydimethylsiloxane (PDMS) microchannel. Between the microchannel’s inlet and outlet, there are ten sensors, divided into two sets: *M*_1_ to *M*_5_ are used for the primary results of the paper, which operate at a frequency of 45.6 MHz, and *N*_1_ to *N*_5_ are used for supportive results, which operate at 38.0 MHz (Fig. 1c, d). The sensors from each set have a *d*_*f*_ varying between 0.7 mm (*M*_1_, *N*_1_) and 1.1 mm (*M*_5_, *N*_5_), and are spaced in intervals of 0.1 mm. The contact area between the fluid and the vibrating surface of the delay line is defined as *w* × *d*_*f*_, where *w* is the channel width. Therefore, the area of *M*_5_ (and *N*_5_) is 1.6 times greater than *M*_1_ (and *N*_1_), such that the dependence of slip on the contact area can be investigated. See Methods for fabrication details.

Seven different water-glycerol solutions with the mass fraction of glycerol ranging from 0 to 0.6 have been used in the experiments. As described in Table S1 in the Supplementary Information, the increase in the mass fraction of glycerol increases the solution’s density from 998 to 1154 kg^.^m^−3^, and the solution’s viscosity from 1 to 10.8 mPa^.^s. Using these seven solutions, the increase in fluid pressure as a result of the increase in density and the increase in fluid shear stress due to the increase in viscosity can be studied. To assess the effect of local pressure on slip due to the acousto-fluidic friction, we evaluated the slip with and without a pressure drop along the channel length, as described in Fig. 1e, f. Having sensors at different positions in the channel allows us to isolate the impact of the local pressure from the shear stress in the measurement of the slip coefficient using both flow directions, *D*_1_ and *D*_2_ (Fig. 1d–f).

### Acoustic loss and the slip coefficient

**Fig. 2:**
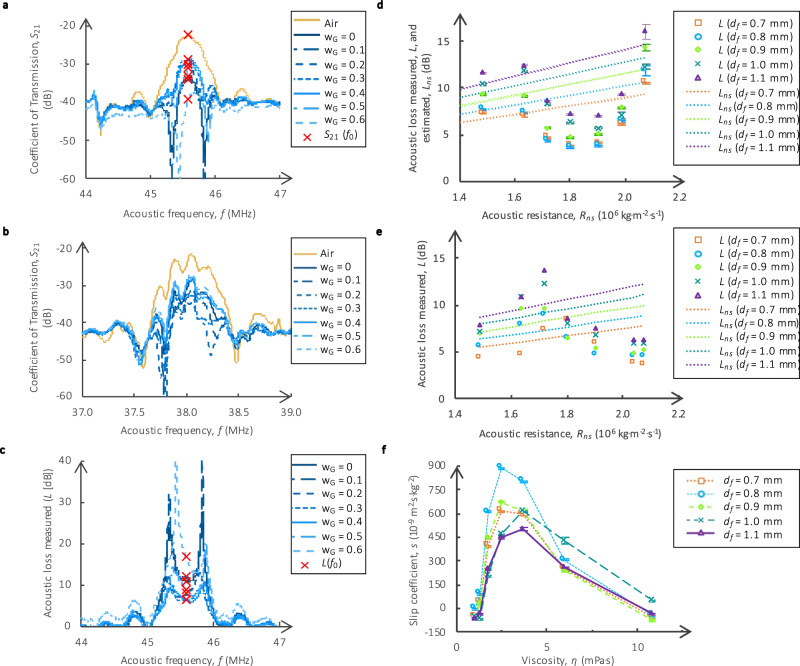
Transmission scattering parameter, S21, and acoustic loss, L, measurements. **a** *S*_21_ measurements for different solutions as a function of the acoustic wave frequency using sensor *M*_3_. The red x marker represents the *S*_21_ value at 45.6 MHz. All *S*_21_ values are expressed in decibels dB, with a drive power of 1 mW. **b** *S*_21_ measurements for different solutions as a function of the acoustic wave frequency using sensor *N*_3_. **c** Experimental acoustic loss, *L*, of different solutions as a function of the acoustic wave frequency using sensor *M*_3_. The red x marker represents the *L* value at 45.6 MHz, which is the chosen frequency to study the impact of flow rate and viscosity on *L*. **d** Acoustic loss as a function of the acoustic resistance of the fluids. *L* is plotted for the five sensors (*M*_1_ to *M*_5_) with different *d*_*f*_ (0.7– 1.1 mm). The expected acoustic loss without slip, *L*_*ns*_, for each *d*_*f*_ is also represented with dashed lines. Since *L* is lower than *L*_*ns*_ for *R*_*ns*_ between 1.67 $\times$ 10^6^ kg^.^m^−2.^s^−1^ and 2 $\times$ 10^6^ kg^.^m^−2.^s^−1^ (i.e., *η* between 1.76 and 6 mPa^.^s), there is slip at the fluid–solid interface for these viscosities. **e** Acoustic loss as a function of the acoustic resistance of the fluids. *L* is plotted for the five sensors (*N*_1_ to *N*_5_) with different *d*_*f*_ (0.7–1.1 mm). Since *L* is lower than *L*_*ns*_ for *R*_*ns*_ above 1.77 $\times$ 10^6^ kg^.^m^−2.^s^−1^ (i.e., *η* above 2.50 mPa^.^s and 6 mPa^.^s), there is slip at the fluid–solid interface for these viscosities. **f** Slip coefficient *s* for the five sensors (*M*_1_ to *M*_5_). For *η* between 1.76 and 6 mPa^.^*s*, *s* is positive. This data confirms that the water-glycerol solutions, with a viscosity *η* between 1.76 and 6 mPa^.^s, exhibit slip. For *η* $\gtrsim$ 5 mPa^.^s, *s* decreases because with the increase in *ρ* the viscous shear stress needed to generate the slip also increases. For *η* ≈ 10.8 mPa^.^s, the slip coefficient is below 0, thus the viscous shear stress is not sufficient to generate slip.

The magnitude of the transmission coefficient between the receiver and the emitter, *S*_21_, is measured in decibels (dB) from 44 to 47 MHz for *M*_1_ to *M*_5_, and from 37 to 39 MHz for *N*_1_ to *N*_5_ using a vector network analyzer (VNA). The central frequency of the acoustic delay line is 45.6 MHz for *M*_1_ to *M*_5_ and 38.0 MHz for *N*_1_ to *N*_5_ as shown in Fig. 2a, b. These frequencies are used to compare the *S*_21_ amplitude of the five sensors in each set (*M* and *N*) with each of the seven solutions. By comparing the *S*_21_ amplitude at the same acoustic frequency for a given sensor set, we compare the sensors at the same acoustic shear rate generated from the acoustic wave. The two sets generate different acoustic shear rates because their operating frequencies are different. The electrical power measured at the receiver of each sensor depends not only on the amount of acoustic energy transferred into the fluid but also on the electrical losses in the transducers and delay line. To isolate the acoustic loss from the electrical losses, we define the acoustic loss in the fluid, *L*, as the difference between the amplitude of *S*_21_ in air and in the fluid (Fig. 2c). Air is used as the reference medium because the acoustic losses in air are expected to be negligible compared to the ones in water-glycerol solutions (Table S1, Supplementary Information). The acoustic loss in the fluid, *L*, depends on: (1) the acoustic resistance in the fluid, *R*; (2) the acoustic resistance in air, *R*_*a*_; (3) the acoustic resistance in the piezoelectric substrate, *R*_*c*_; and (4) the wavelength of the acoustic wave, *λ*, as described by Eqs. (1)–(3). The acoustic loss per unit length^27,28^ when either fluid or air is in the microfluidic channel is described by *α* and *α*_*a*_, respectively.

1 $$L=\,\left(\alpha -{\alpha }_{a}\right){d}_{f}$$

2 $$\alpha =\frac{20}{{{{{{\rm{ln}}}}}}\left(10\right)}\frac{R}{{R}_{c}}\frac{1}{\lambda }$$

3 $${\alpha }_{a}=\frac{20}{{{{{{\rm{ln}}}}}}\left(10\right)}\frac{{R}_{a}}{{R}_{c}}\frac{1}{\lambda }$$

Without slip, *R* is described by *R*_*ns*_, the product of the medium’s density, *ρ*, and the acoustic speed, *c*, as shown in Eq. (4). From the work of McHale et al.^21^, when slip occurs, the acoustic resistance of the fluid, *R*, becomes *R*_*s*_, which is dependent on *R*_*ns*_ and the slip coefficient, *s*, as defined in Eq. (4).

4 $$R=\left\{\begin{array}{c}{R}_{{ns}}=\,\rho c,\,{{{{{{\mathrm{if}}}}}}}\,{{{{{{\mathrm{no}}}}}}}\,{{{{{{\mathrm{slip}}}}}}}\hfill\\ {R}_{s}=\,\frac{{R}_{{ns}}}{1+s{R}_{{ns}}},\,s \; > \; 0\,{{{{{{\mathrm{if}}}}}}}\,{{{{{{\mathrm{slip}}}}}}}\,\end{array}\right.\,$$

When there is no-slip, *R* increases linearly with the fluid density. From Eqs. (1) and (2), this increase in the acoustic resistance leads to a linear increase in the acoustic loss into the fluid. Therefore, we expect that *L* increases linearly by increasing the mass fraction of glycerol, *w*_*G*_. Once there is slip, indicated by *s* > 0 in Eq. (4), the acoustic resistance of the fluid, *R*, is lower than *R*_*ns*_. Thus, the measured acoustic loss, *L*, should be smaller than without slip. Deviations from the expected acoustic resistance under no-slip conditions, *R*_*ns*_, provide an indication that slip is present at the fluid–solid interface^21^.

### Slip from acousto-fluidic friction

Figure 2d presents the measured acoustic loss in the fluid, *L*, for the five sensors, *M*_1_ to *M*_5_, with the seven water-glycerol solutions and no flow. To compare the loss in the different solutions, the solutions are characterized by their *R*_*ns*_ values. To highlight the presence of slip, which is indicated by *L* < *L*_*ns*_, *L*_*ns*_ is also plotted in Fig. 2d. The measured acoustic loss, *L*, is smaller than *L*_*ns*_ for *R*_*ns*_ between 1.67 × 10^6^ kg^.^m^−2.^s^−1^ and 2 × 10^6 ^kg^.^m^−2.^s^−1^, which corresponds to water-glycerol solutions with a mass fraction of glycerol between 0.2 and 0.5 and a viscosity between 1.76 mPa^.^s and 6 mPa^.^s (Table S1, Supplementary Information), indicating that there is slip at the fluid–solid interface for these values of *R*_*ns*_.

While the observed reduction in acoustic coupling is considered a clear signature of slip^21^, we have explored other possible causes. One possibility is that the aqueous glycerol solutions had a lower density than expected, for example, due to incomplete mixing. Another possibility is that air trapped between the liquid and substrate could reduce their interaction. The measurements in Fig. 2d were repeated on different days, with different batches of glycerol solutions, and with different sensors. The same results were found in all cases. Therefore, the above-considered causes are highly unlikely. As a result, the most likely explanation for the acoustic loss is that the liquid slips due to the surface acoustic waves as proposed by McHale et al.^21^.

The possible causes of slip at the fluid–solid interface are the shear stress^13–15^, wall roughness^16,17^, and surface tension^17,18^. Since we use the same channel to study the different water-glycerol solutions, the wall roughness does not change and therefore, cannot be the cause of the slip behavior. However, it is reasonable to consider whether surface tension plays a role in acoustic loss. The contact angle, *θ*, is commonly used to determine the surface tension when there are three materials interacting within a microfluidic channel: typically air, liquid, and solid. In our case, there is only a liquid-solid interface. Therefore, it is more appropriate to look at the reversible adhesion energy of a liquid on a surface, *W*_*SL*_^31,32^, as shown in Eq. (5), where *γ*_*S*_, *γ*_*L*_, and *γ*_*SL*_ are the surface tensions of the substrate, liquid, and the interface between the liquid and the substrate, respectively. Equation (5) does not consider the pressure applied by the liquid on the surface. Among the three surface tensions (*γ*_*S*_, *γ*_*L*,_ *γ*_*SL*_), *γ*_*SL*_ is the most difficult to measure, but can be calculated using *θ* and *γ*_*L*_^17,33^. The interface affecting the acoustic wave is between the silicon oxide surface and the liquid. We measured the contact angle between this surface and the liquid to calculate *γ*_*SL*_, as reported in Table S2 in Supplementary Information. From this, the adhesion energy of water on silicon oxide is calculated to be 77 mJ^.^m^−2^, and for a water-glycerol mixture with *w*_*G*_ = 0.6 it is 84 mJ^.^m^−2^ (Table S2, Supplementary Information).

5 $${W}_{{SL}}={\gamma }_{S}+{\gamma }_{L}-{\gamma }_{{SL}}={\gamma }_{L}({{\cos }}\left(\theta \right)+1)$$

The adhesion energy increases with the increase in mass fraction of glycerol. Therefore, the adhesion energy does not contribute to changes in the acoustic loss since it would have to decrease for increasing mass fraction to explain the data in Fig. 2d. Having exhausted the other possible causes for slip, it is clear that it is most likely due to the magnitude of the shear stress, as described next.

### Critical shear stress from static to slip regime

To further confirm the results, we performed the same experiments on sensors *N*_*1*_ to *N*_*5*_ (Fig. 2e). These sensors operate at a lower acoustic frequency than *M*_1_ to *M*_5_, thus generating a lower acoustic shear rate than *M*_1_ to *M*_5_. Similar to results from *M*_*1*_ to *M*_*5*_, the acoustic loss for *N*_*1*_ to *N*_*5*_ increases and then decreases for increasing values of *R*_*ns*_. The onset of slip occurs at an acoustic impedance of 1.77 × 10^6^ kg^.^m^−2.^s^−1^ for *N*_*1*_ to *N*_*5*_, which corresponds to a mass fraction of glycerol of 0.3 and a viscosity of 2.50 mPa^.^s (Table S1, Supplementary Information). At this same acoustic impedance (i.e., same viscosity), the sensors *M*_*1*_ to *M*_*5*_ have already begun to slip on the surface acoustic waves. The delayed onset of slip for sensors *N*_*1*_ to *N*_*5*_ in terms of viscosity is due to the lower acoustic frequency, which yields a lower acoustic velocity. The lower acoustic velocity for *N*_*1*_ to *N*_*5*_ requires a higher viscosity to achieve the same acoustic shear stress as the sensors *M*_*1*_ to *M*_*5*_ at the onset of slip, demonstrating that the slip behavior is directly related to a threshold for the shear stress^16^.

The results in Fig. 2d, e show that the critical shear stress for generating slip is reached once the viscosity is higher than 1.76 mPa^.^s for *M*_*1*_ to *M*_*5*_ and 2.50 mPa^.^s for *N*_*1*_ to *N*_*5*_. In solid mechanics is usually used the resultant force placed at the center of mass, which is not an actual applied force. The resultant of the shear stress also presents a threshold of motion in the Amontons-Coulomb laws of dry friction, known as the static friction threshold, and represents the transition between static friction (no-slip) and kinetic friction (slip). For dry friction, the slip velocity is constant once the slip occurs. In microfluidics, the slip length, *β*, is more commonly used and is a function of the slip velocity and the viscous pressure drop (Δ*P/η* or *τ*, see Supplementary Information). Similar to dry friction, in microfluidics, the slip length only increases and eventually reaches a plateau for increasing shear stress^13,14^. Figure 2f presents the slip coefficient, *s*, for the five sensors, *M*_*1*_ to *M*_*5*_, with the seven solutions of water-glycerol characterized by their viscosity, *η*, where *s* is calculated using Eqs. (1)–(4) and the data in Fig. 2d. All sensors show the same trend and peak near the same value of *η*. Thus, the generation of slip is independent of the contact area, similar to Amontons-Coulomb’s second law of friction. For viscosities higher than the critical viscosity, 1.76 mPa^.^s, the slip coefficient increases for *η* $\lesssim$ 5 mPa^.^s, and then, decreases for *η* $\gtrsim$ 5 mPa^.^s to reach 0 at *η* ≈ 10.8 mPa^.^s. The slip coefficient does not reach a plateau, and to our knowledge, this result is contrary to all previous results on acousto-fluidic slip. However, unlike previous studies^13,14^ on the effect of the shear stress on the slip length, the increase in shear stress is not continuous since it is controlled using solutions of varying viscosity, rather than increasing the flow rate with a single solution. Furthermore, by changing the solution, we not only change the viscosity but also the density. Therefore, we change the force on the fluid–solid interface since the pressure of the fluid increases with the increase in density. In the case of an increase in density, Geng et al.^34^ have shown it reduces the slip length. This is an interesting fact as the critical shear stress may depend on the mass loading, similar to the Amontons-Coulomb laws of dry friction. In the following sections, we study the impact of the fluid pressure on the critical shear stress, and their effect on the slip dynamics.

### Critical shear stress and pressure coupling in acousto-fluidic slip

**Fig. 3:**
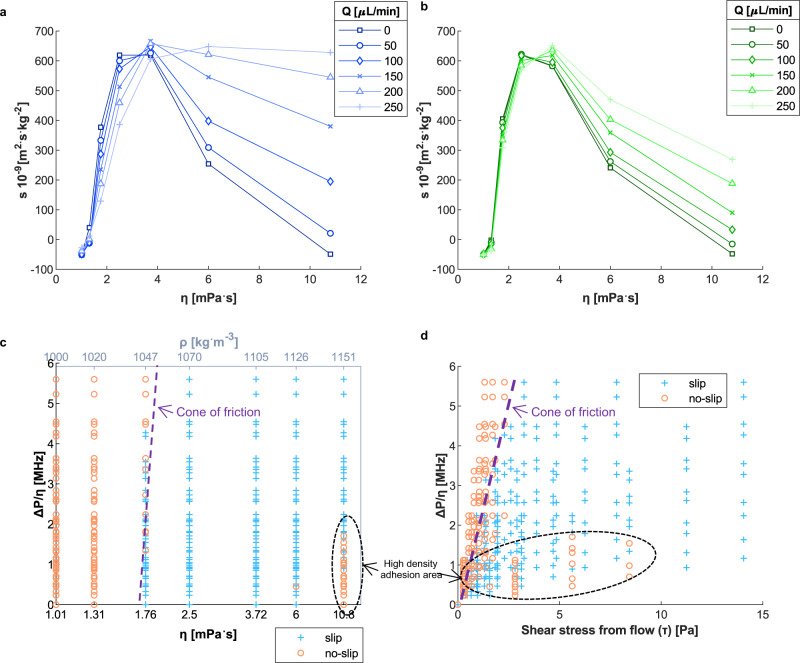
Coupling between pressure and shear stress for acousto-fluidic slip. **a, b** Slip coefficient *s* of *M*_1_ for different flow rates, *Q*, in the direction *D*_1_ (**a**) and *D*_2_ (**b**) as a function of the fluid viscosity, *η*. The amplitude of *s* depends on the flow direction. Therefore, the slip depends on the pressure. **c**, **d** Shear stress and pressure conditions generating acousto-fluidic slip using all sensors *M*_1_ to *M*_5_. **c** Focus on the acoustic shear stress. Most of the no-slip conditions with regard to flow pressure and acoustic shear are concentrated in a cone that is analogous to the friction cone. This friction cone separates the area where the fluid pressure exceeds the acoustic shear stress (no-slip red circle markers), and the area where the acoustic shear stress surpasses the fluid pressure (slip blue plus sign markers). A second adhesion area (black oval) at *η* = 10.8 mPa^.^s indicates that the pressure due to the fluid mass surpasses the acoustic shear stress. **d** Focus on the flow shear stress. The friction cone separates the flow pressure-shear conditions generating adhesion (orange circles) from the conditions generating slip (blue plus sign). The pressure related to the fluid mass is compensated by the increase in shear stress from flow, switching from an adhesion regime at high viscosity (black circle) to a slip regime. The shear stress transition threshold is ≈ 10 Pa.

In the above results, the fluid is static, so the pressure is the same everywhere in the channel. However, under flow conditions, the pressure applied to the acoustic waves varies with the location of the sensor in the channel (Fig. 1e, f), but the shear stress is the same throughout the channel. Previous work has not investigated the coupling between the local pressure and the shear stress for an interfacial slip at the fluid–solid boundary in acousto-fluidic systems. As a result, flow measurements were performed to, first, determine whether pressure affects acousto-fluidic slip and, second, whether the critical shear stress depends on pressure similar to the Amontons-Coulomb laws. In these experiments, we reversed the flow direction, from *D*_1_ to *D*_2_, which changes the distance between the outlet and the sensor (Fig. 1d). The slip coefficient for sensor *M*_1_ as a function of viscosity and flow rate is shown in Fig. 3a and b for flow directions *D*_1_ and *D*_2_, respectively. For both *D*_1_ and *D*_2_, the slip coefficient is clearly affected by the flow rate, where *s* decreases with the increase in flow rate for *η* $\lesssim$ 5 mPa^.^s, but increases with the increase in flow rate for *η* $\gtrsim$ 5 mPa^.^s. However, the variation in *s* due to the flow rate is much larger when the fluid flows in the *D*_1_ direction than for *D*_2_. Due to its location in the channel, the pressure applied on *M*_1_ is much higher for *D*_1_ than *D*_2_ (Fig. 1e, f). This observation clearly shows that the slip behavior depends on the local fluid pressure. In the Supplementary Information, Fig. S2 shows the slip coefficient for different flow rates and both flow directions for all sensors, *M*_1_ to *M*_5_. Since *M*_5_ is located towards the end of the channel, like *M*_1_, its response is similar to *M*_1_. However, *M*_3_ is placed at the center of the channel, so its response is not affected by the change of flow direction. These results demonstrate that slip is dependent on pressure, or the normal force, as described by the Amontons-Coulomb laws.

The Amontons-Coulomb laws state that the static friction threshold is proportional to the resultant of the normal forces. Applying this law to our system is equivalent to a coupling between pressure (i.e., normal force), *P*, and critical shear stress (i.e., tangential force), *τ*, at the fluid–solid interface. When looking at the relationship between *P* and *τ*, there is a linear boundary between the no-slip and slip regions defined by *τ* = *µP*, where *µ* is the coefficient of friction and the boundary is called the cone of friction^28,29^. This boundary defines the critical shear stress for slip depending on the pressure applied by the solid. To further understand the fluid–solid interaction in this context, we now look at how slip depends on *P* and *τ*. Under flow conditions, the increase in flow rate, *Q*, generates an increase in flow shear stress, *τ*, as expressed in Eq. (6) with channel width, *w*, and channel height, *h*. From Eq. (7), the flow pressure drop, Δ*P*, between a distance *x* from the outlet and the outlet increases with *x*, *τ*, and *η*^17^. Due to the dependence on *x*, reversing the flow direction doubles the dataset for *τ* and *P* since the amplitude of the local fluid pressure on the sensors (Fig. 1e, f) changes with *x* but the shear stress does not.

6 $$\tau =\frac{6}{w{h}^{2}}Q$$

7 $$\frac{\triangle P}{\,\eta }=\,\frac{2x}{h}\,\tau$$

For the different *Q* and *x*, the resulting (*τ,P*) values were calculated using Eqs. (6)-(7) and correlated with the measured acoustic loss for *M*_1_ to *M*_5_, as shown in Fig. 3c,d. For each set of (*Q*,*x*) and (*τ,P*), we measured the acoustic loss and extracted the slip coefficient. Based on this data, in Fig. 3c, d, the fluid is considered to slip (‘’ in blue) when *s* is over 150 ${{{{{\boldsymbol{\times}}}}}}$10^−9^ m^2.^s^.^kg^−1^; otherwise, the fluid adheres to the piezoelectric substrate (‘’ in orange). The slip coefficient threshold value between the adhesion and slip regimes is estimated based on the confidence interval of no-slip (details in Supplementary Information). The complete dataset of *s* for varying flow rate using all sensors, before the correlation to (*τ,P*) values, is shown in the Supplementary Information (Fig. S2).

Pressure depends on the density and the flow rate. However, here we present the pressure, normalized by the viscosity (Δ*P/η*, *y*-axis in Fig. 3c, d), resulting from the flow and ignore the mass contribution since the effects of density and viscosity cannot be decoupled for the water-glycerol solutions. In addition, there are two sources of shear stress, acoustic shear stress, and flow shear stress. Therefore, the effects from the acoustic shear stress (Fig. 3c) and the flow shear stress, $\tau$ (Fig. 3d) have been presented separately. In Fig. 3c, the acoustic shear stress on the *x*-axis is represented in terms of the viscosity since the acoustic shear stress is proportional to the viscosity multiplied by the acoustic frequency, which is fixed at 45.6 MHz in the experiments for *M*_1_ to *M*_5_.

In Fig. 3c, d, most of the no-slip pressure-shear stress conditions are concentrated in a region that is similar to the cone of friction used to describe dynamic dry friction^28,29^. This cone of friction separates the area where the fluid pressure exceeds the shear stress (no-slip markers), and the area where the shear stress surpasses the fluid pressure (slip markers). However, for *η* = 10.8 mPa^.^s (Fig. 3a, c), the fluid does not slip at low Δ*P/η*. This second no-slip area (circled in black) does not exist in dry friction. As mentioned previously, the increase in fluid density (top *x*-axis Fig. 3c) when increasing the fluid viscosity (bottom *x*-axis Fig. 3c) may generate a second adhesion area due to mass loading. We note that in this second adhesion area, the pressure related to the fluid mass surpasses the acoustic shear stress. In Fig. 3d, this second adhesion area is confined in a low-*P* and low-$\tau$ area. This means that, with the increase in flow rate, the flow shear stress increases to the point that the fluid starts slipping. This phenomenon is also shown in Fig. 3a, b for *M*_1_. The transition is observed for *τ* ≈ 10 Pa. This result confirms that the second adhesion area is related to the fluid mass surpassing the acoustic shear stress, and that the pressure from the fluid mass can be compensated by increasing the shear stress with the flow. Thus, the critical shear stress to generate the slip depends on the pressure applied at the interface as described in the Amontons-Coulomb laws. The slope of the cones of friction shown in Fig. 3c, d provides a simple relationship between pressure and shear stress that defines the no-slip/slip boundary and can be used to improve the design of acousto-microfluidic devices.

### Slip regime in acousto-fluidic friction

The Amontons-Coulomb laws state that once slip occurs, the resultant of the tangential forces (*τ* in our case) is proportional to the resultant of the normal forces (*P* in our case) and that the effort needed to maintain the slip is smaller than the effort needed to generate the slip (i.e., constant slip velocity). In microchannels, with or without slip, Δ*P/η* and *τ* are coupled as expressed in Eq. (7), where *x* is the distance between the sensor and the outlet and *h* is the channel height. In Fig. 2f, without flow, the slip coefficient increases and, instead of reaching a plateau, it then decreases due to mass loading with the increase of the acousto-fluidic friction. When there is flow in the channel, the effort to generate the slip comes also from the flow shear stress. However, when using a syringe pump, the flow shear stress and pressure depend on the slip length^35^ (Table S2, Supplementary Information), while the flow rate is constant. Therefore, to avoid confusion, we use the term “flow effort” to express the value of Δ*P/η* without slip.

**Fig. 4:**
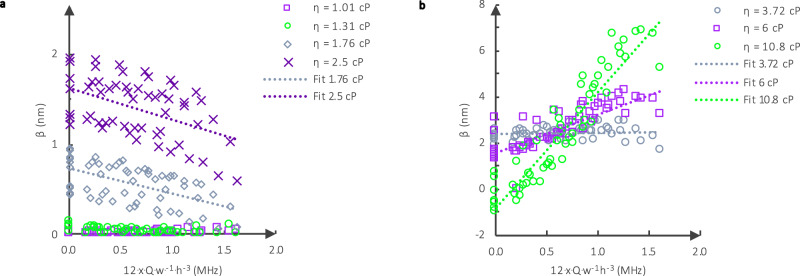
Experimental determination of the slip length, β. **a** Case 1: Low viscosity, *η* < 3 mPa^.^s. Slip length for different solutions characterized by their viscosities as a function of the flow effort (12^.^x^.^Q/(w^.^h^3^). *β* decreases with the increase in the viscous pressure. **b** Case 2: Medium viscosity, 3 mPa^.^s < *η* ≤ 10.8 mPa^.^s. Slip length for different solutions as a function of the flow effort 12^.^x^.^Q/(w^.^h^3^). *β* increases and reaches a maximum. The slip length is plotted independent of *d*_*f*_ and the data show that *β* does not depend on the contact area.

To further study the evolution of the slip length in the slip regime, we evaluated *β* as a function of the effort from the flow rate. Ellis et al.^22^ have shown that the slip length is related to the slip coefficient by *β = ηs*. In Fig. 4, the calculated slip length based on the loss data for the five sensors, *M*_1_ to *M*_5_, with a varying flow rate in both directions *D*_*1*_ and *D*_*2*_ is represented as a function of the flow effort for the different water-glycerol solutions. The slip length is on the order of a few nanometers for all measurement conditions, which matches with previous results^22^. For *η* ${{{{{\boldsymbol{\lesssim }}}}}}$ 3 mPa^.^s (Fig. 4a), the slip length decreases with the increase in flow effort, while for 3.72 mPa^.^s ${{{{{\boldsymbol{\gtrsim }}}}}}\,$*η* ${{{{{\boldsymbol{\gtrsim }}}}}}$ 10.8 mPa^.^s (Fig. 4b), *β* increases with the increase in flow effort, and reaches a maximum. The increase in slip length happens during the transition between the static and slip regimes. Whereas the decrease in the slip length happens in the slip regime. Therefore, contrary to dry friction, in the slip regime, the slip length is not constant but decreases with the increase in shear effort. Moreover, in the slip regime, the slope of *β* increases with the increase in viscosity or density, which means that more viscous or more dense fluids have better adhesion to the surface at higher flow rates.

Finally, the fact that the slip length decreases with the flow effort, which would be Δ*P/η* without slip, makes the slip length dependent on the parameter *x* (Eq. (7)). This shows that the slip length is not constant throughout the channel at a constant flow rate. For example, for *η* = 6 mPa^.^s and *Q* = 150 μL^.^min^−1^, the slip coefficient is smaller at the outlet than at the inlet (Fig. 3a, b). Typically, the slip length, *β*, is considered constant with respect to flow rate^11,12^. However, some experiments^13–15^ have shown that the shear stress increases the slip^13,14^, while others have found that the shear stress decreases the slip^15^, and a third group found that the pressure decreases the slip^11,12^. Here, we have experimentally demonstrated that these behaviors are not contradictory and are all possible within a single acousto-fluidic sensor when the pressure and shear stress are varied through changes in the flow rate, viscosity, and location of the sensor in the channel.

## Discussion

From our assumption that water-glycerol solutions slip at a fluid-surface acoustic wave interface, we have shown that the slip happens once the shear stress surpasses a critical value. Our experiments confirm that the critical shear stress depends on the pressure applied by the fluid at the interface. When the fluid is static, the slip transition depends on the fluid density and viscosity, and when there is flow, the slip transition depends on the local flow pressure and shear stress. Moreover, we have shown that the critical shear stress does not depend on the area of contact. We have also shown that the acoustic loss, the slip coefficient, and the slip length depend on the combination of the viscous shear stress and the fluid pressure generated by the flow and applied at the interface. This behavior closely resembles the dynamic dry friction described by the Amontons-Coulomb laws, which has not previously been observed in acousto-microfluidic systems. Our experiments show that, in the pressure-shear stress graph, the no-slip area for acousto-fluidic friction is confined in a cone that is analogous to the cone of friction found in dry friction. Furthermore, this phenomenon leads to a slip length that varies along the channel length. This behavior was found to be highly repeatable in our sensors across different sensor geometries. Future work will focus on an analytical description of these complex slip dynamics at the fluid–solid interface.

The slip behavior is expected to have a profound effect on the performance of acousto-microfluidic sensors and actuators. For example, surface acoustic wave-based devices for proteomic analysis isolate plasma extracellular vesicles^4^ using the acoustic radiation force, which depends on the acoustic pressure. In this paper, the slip was studied for ranges of density and viscosity similar to those found in biofluids, including human blood plasma. The natural variance in viscosity for human plasma^36,37^, between 1.15 and 1.8 mPa^.^s, lies between the adhesion and slip regimes for our sensors (Fig. 3c). As shown in Fig. 3, slip may appear depending on the flow rate and a patient’s plasma viscosity. As shown in Fig. 2c, d, when slip is present, the acoustic energy transferred into the fluid is lower than anticipated. For example, for a viscosity of 1.7 mPa^.^s, the calculated acoustic radiation force, *L*_*ns*_ = 7.7 dB in Fig. 2d, was 1.7-times higher on a linear scale compared to the measured value, *L*_*s*_ = 4.4 dB. Whereas at 1.3 mPa^.^s, there is no-slip, thus the calculated value is similar to the measured value. Therefore, the acoustic radiation force will vary from patient to patient due to both the viscosity and the acousto-fluidic slip, likely yielding anomalous results from the device.

Another example of the importance of our findings is in the use of surface acoustic waves for cell sorting in whole blood^1^. For the typical range of flow rate, and also shear rate, the natural variance in whole blood viscosity is between 3 and 6 mPa^.^s^38^. In our sensors, the fluid slips in this range of viscosity (Fig. 3c, d). We demonstrated that the amplitude of the slip length, as well as the acoustic radiation force, depends not only on the viscosity but also on the pressure and the shear stress. This means that the sorting quality is dependent on the whole blood flow rate and the position of the acoustic actuator within the channel (Fig. 4a, b). Thus, the complex slip dynamics described here should be considered when designing future acousto-microfluidic devices to achieve reliable and accurate performance that will advance future measurements in biology and medicine.

## Methods

### Glycerol-water solutions

Glycerol solutions were prepared by weighing glycerol anhydrous for each of the mass fractions, and then the final mass for each solution was reached by adding pure water. The mass fractions, *w*_*G*_, for the glycerol solutions prepared were: 0 (pure water), 0.1, 0.2, 0.3, 0.4, 0.5, and 0.6. Their properties are reported in Table S1 in the Supplementary Information.

### Fabrication of the acousto-microfluidic sensor

Piezoelectric 128° XY-cut black lithium niobate (LiNbO_3_) wafers with a diameter of 100 mm and thickness of 500 μm were used to fabricate the surface acoustic wave devices. First, the wafers were cleaned by soaking in acetone for 5 min, then in isopropanol for another 5 min, and finally in deionized (DI) water for at least 5 min. The wafer surface was activated by an argon plasma and an oxygen plasma using a reactive ion etching chamber. These steps improved the adhesion of the lift-off resist on the wafer. The argon plasma activation was done under a pressure of 33.33 kPa (=250 mT), 50 W, and a flow of 15 cm^3.^min^−1^ (=15 sccm) during a 20 s period. The oxygen plasma activation was carried out at 26.66 kPa (=200 mT), 200 W, and a flow of 50 cm^3.^min^−1^ for a period of 20 s. The lift-off resist was deposited on the wafer using a spin-coater. After spin-coating with lift-off resist, the wafer was placed on a hot plate, at room temperature (~20 °C). The wafer was then heated up to the optimal soft-baked temperature and then cooled down using a ramp of 5 °C/min. Ramping the temperature up and down at these rates prevented the lithium niobate wafer from breaking due to the pyroelectric effect. The photoresist was spin-coated on top of the lift-off resist to get a photoresist thickness of 1.3 μm. A soft bake step was then carried out. Then, the wafer was electrically discharged by short-circuiting its two sides. Using a mask aligner, the interdigitated transducer design was transferred into the photoresist by UV exposure of the wafer through a chrome mask. The photoresist was developed by the photoresist’s developer. The wafer was then rinsed in DI water and blow-dried using nitrogen gas. The wafer surface was activated by using an argon plasma followed by an oxygen plasma, using the same conditions as described above. This surface activation helped the titanium to stick on the lithium niobate. An adhesion layer of titanium (10 nm thick) followed by a layer of gold (90 nm thick) was deposited on top of the lithium niobate using an electron beam evaporator. The resists were then lifted off in a resist remover for 24 h. The wafer was finally cleaned using a three-step process: immersion in acetone for 5 min, then in isopropanol for 5 min, and finally in DI water for at least 5 min. The wafer was then blow-dried with nitrogen gas. A resist was then spin-coated on top of the wafer to protect the wafer from dust. The wafer was diced into 3 cm $\times$ 3 cm chips. The resist was cleaned again by immersion in the same 3 solvents above mentioned: acetone (5 min), isopropanol (5 min), and DI water (5 min). The wafer was then blow-dried with nitrogen gas. Resist residue was removed using argon plasma and oxygen plasma. The oxygen plasma can be used for up to 2 min. To improve the adhesion of the PDMS microchannel on top of the lithium niobate processed substrate, a silicon oxide layer of 100 nm thick was sputtered on top of the wafer, only where the PDMS would be in contact with the substrate. A silicon wafer was used as the mold for the PDMS microchannel network. To fabricate the mold, the silicon wafer was dehydrated for 5 min at 200 °C. Then, a photoresist was spin-coated on the silicon wafer to obtain a final thickness of around 80 μm. The silicon wafer was soft-baked, on a hot plate. The microchannel design was transferred into the resist using a chrome mask and UV exposure. The wafer was then hard-baked and developed. The wafer was then rinsed for 1 min in isopropanol. The two parts of the silicone elastomer kit were mixed using a 10:1 mix ratio. The mix was then de-gassed for 20 min before pouring it on the wafer. The PDMS thickness was between 3 and 5 mm. The wafer with the uncured PDMS was placed for an hour in a desiccator connected to a vacuum line for de-gassing. Then, the PDMS was cured for 8 h at 80 °C in an oven. The silicon oxide on the substrate surface was activated using an argon plasma and an oxygen plasma, the same process described above. The PDMS microchannel was peeled off the mold and cut to fit the silicon oxide area on the substrate. Inlets were made in the PDMS using a hole punch. The PDMS microchannel was then aligned and put in contact with the substrate.

### Interfaces and data collection

The medium we used as a reference for measuring the *S*_21_ response of the sensors was air. The emitter and the receiver were connected to a vector network analyzer by using a probe station. The vector network analyzer (VNA) was configured to measure the magnitude in decibels (dB), with a drive power of 1 mW, and the phase between the output and the input. The amplitude ratio, also called the acoustic loss, was measured on a frequency interval from 44 to 47 MHz with a frequency spacing of 1.874 kHz. Fluid was loaded into the channel using a syringe pump. The inlet was connected to the syringe with a solvent compatible and flexible tube. The outlet was connected to a waste container with a solvent compatible and flexible tube. The two tubes for the inlet and outlet had the same length. The loading flow rate was between 10 and 50 μL/min. Once loaded the flow was stopped. The fluid rested for 5 min. Then, using the same configuration as for the reference measurement, the acoustic losses were measured using a VNA. The flow rate was then increased by 50 μL/min using a syringe pump. The measurement was done after waiting between 20 s to 5 min, depending on the fluid viscosity. The rest time allowed the fluid to reach its steady-state. This step was repeated until the flow rate reached 250 μL/min. Then, the flow rate was stopped. The full measurement was repeated two more times after a resting time of 5 min between each measurement. This was done for the 10 sensors on the chip. Then, the inlet and the outlet were exchanged to revert the flow direction. The full measurement was repeated as described above. At the end of the experiment, the fluid was removed with air using a syringe pump.

## Supplementary information

Supplementary Information

## Data Availability

The datasets generated and/or analyzed during the current study are available at the following link: 10.18434/mds2-2540.
