# Supplementary material for: Amontons-Coulomb-like slip dynamics in acousto-microfluidics
Source: Nat Commun. 2022 Mar 22;13:1429. doi: 10.1038/s41467-022-28823-6 (PMC8941090; doi:10.1038/s41467-022-28823-6)
Supplement: Supplementary file 1 — Supplementary Information [file 41467_2022_28823_MOESM1_ESM.pdf]

Supplementary Information

Amontons-Coulomb-like slip dynamics in acousto-microfluidics

Aurore Quelennec[1], Jason J. Gorman[1], and Darwin R. Reyes[1*]

[1]National Institute of Standards and Technology, Gaithersburg, MD 20899 USA

[*]email: darwin.reyes@nist.gov

Glycerol-water solutions. Glycerol solutions were prepared by weighing glycerol anhydrous for each of the mass fractions, and then the final mass for each solution was reached by adding pure water. The mass fractions, w_G, for the glycerol solutions prepared were: 0 (pure water), 0.1, 0.2, 0.3, 0.4, 0.5 and 0.6. Their properties are reported in Table S1 and Table S2. Contact angles were measured for all solutions on silicon oxide deposited on a lithium niobate substrate, as listed in Table S2. Drops of water and 6 glycerol solutions with mass fractions of glycerol in water between 0.1 and 0.6 were placed on the surface, and a side-view picture was taken. The contact angle value was obtained using image processing software. Ten measurements were taken for each solution.

Table S1. Material properties and acoustic loss. The viscosity, η, the density, ρ, the speed of sound in the material, c, the acoustic resistance without slip estimated, R_{ns}, and the acoustic loss estimated per unit length, α, are given for the crystal, PDMS, air and the different water-glycerol solutions studied.

Material		η (mPa·s)	ρ (kg·m^{-3})	c (m·s^{-1})	R_{ns} 10^6 (kg·m^{-2}·s^{-1})	α for M_1 to M_5 (dB/(100µm))	α for N_1 to N_5 (dB/(100µm))
128 XY LiNbO3 with SiO2			4650	3650			
	PDMS		969	1120	1.09	0.69	0.58
	Air	0.02	1	330	0.00	0.00	0.00
Water-glycerol mixtures characterized by their mass fraction of glycerol (w_G)[1-2]	0.00	1.00	998	1490	1.49	0.95	0.79
	0.10	1.31	1022	1543	1.58	1.01	0.84
	0.20	1.76	1047	1595	1.67	1.07	0.89
	0.30	2.50	1073	1648	1.77	1.13	0.94
	0.40	3.72	1099	1701	1.87	1.20	1.00
	0.50	6.00	1126	1754	1.98	1.26	1.05
	0.60	10.80	1154	1806	2.08	1.33	1.11

Table S2. Adhesion energy W_{SL} is calculated using our contact angle, θ, measurement and the surface tension of liquid, γ_L, extracted from the literature[1-5].

Material		γ_L,[1,2] ($mJ \cdot m^{-2}$)	θ (°)	W_{SL} ($mJ \cdot m^{-2}$)
	SiO_2[3]	65		
Water-glycerol mixtures characterized by their mass fraction of glycerol (w_G)	0.00	72	86	77
	0.10	72	85	78
	0.20	71	82	81
	0.30	71	81	82
	0.40	70	81	81
	0.50	69	78	83
	0.60	68	76	84

Velocity profile, slip length and pressure drop in microfluidic channels with and without slip. In microfluidics, the velocity profile at steady state in a channel cross-section (*yz* plane in Fig. S1) along the flow direction (*x*-axis in Fig. S1) has a Poiseuille profile as shown in Fig. S1. Therefore, the velocity is parabolic along the *z* and *y*-coordinates. The geometric parameters of the channel are the height (h) along the y-axis, the width (w) along the z-axis and the length (L) along the x-axis. In our study, since $L \gg w \gg h$, we can neglect the parabolic velocity profile along the z axis. Therefore, we can assume that the fluid velocity, v, depends only on the distance along the y axis. The standard no-slip condition means that the fluid velocity at the wall is zero (Fig. S1a). Alternatively, slip at the wall means that the fluid velocity is non-zero there and is described as V_s (Fig. S1b). Instead of using the slip velocity V_s for analysis of the slip condition, the slip length, β, is more commonly used. As shown in Fig. S1b, β is an artificial distance between the wall of the channel and the hypothetical no-slip wall that can be found by extending the velocity profile into the substrate until $v(y) = 0\ m \cdot s^{-1}$.

The slip length can be expressed by the viscous pressure drop between the position and the outlet, $\Delta P/\eta$, the distance, x, between the position in the channel compared to the outlet, h, and V_s (Eq. (S1)). With or without slip, the relation between $\Delta P/\eta$ and the shear stress on the wall, τ, is the same (Eq. (S2)). When using a syringe pump, the flow rate, Q, is constant with or without slip. However, both $\Delta P/\eta$ and τ values are impacted by the slip. The slip length can be expressed in Eq. (S3) as a function of the slip and the no-slip values of $\Delta P/\eta$, with "*s*" and "*ns*" in subscript for slip and no-slip, respectively. Therefore, the slip reduces the pressure drop in the channel compared to the no-slip pressure. In Fig. 4, we evaluated β as a function of the flow effort, $12 \cdot x \cdot Q/(w \cdot h^3)$, which is $\Delta P/\eta$ without slip.

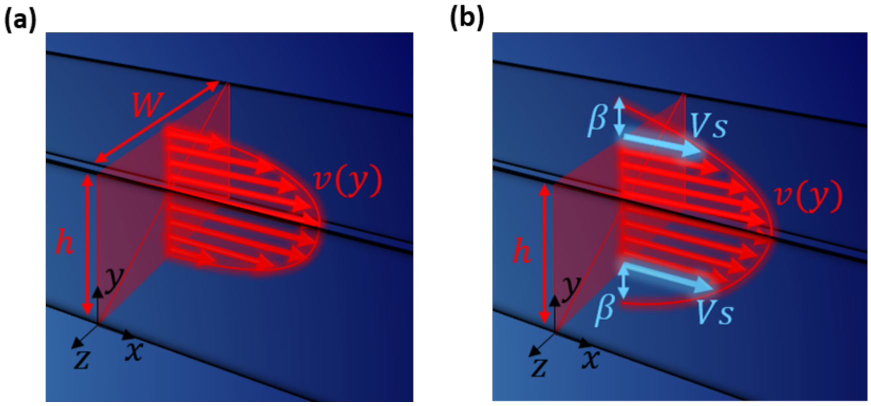

Fig. S1 Velocity profile in a parallelepipedal microfluidic channel. (a) Velocity profile without slip. The velocity at the wall is zero. (b) Velocity profile with slip. The velocity at the wall equals the slip velocity (V_s). The slip length β is the distance between the wall of the channel and the hypothetical no-slip plane located in the substrate that is described by the zero-point of the velocity profile.

$$\beta = \frac{2\,\eta\,x\;V_s}{\Delta P\;h} \tag{S1}$$

$$\frac{\Delta P}{\eta} = \frac{2x}{h}\,\tau \tag{S2}$$

$$\left(\frac{\Delta P}{\eta}\right)_{ns} = \frac{12x}{wh^3}Q = \left(\frac{\Delta P}{\eta}\right)_s\left(1+6\frac{\beta}{h}\right) \tag{S3}$$

Slip coefficient, *s*, for different flow rates. The slip coefficient is shown as a function of viscosity and flow rate, and flow along direction D_1 in Figs. S2a-e for sensors M_1 to M_5, respectively. Similarly, the slip coefficient for flow along direction D2 are shown in Figs. S2f-j for sensors M_1 to M_5, respectively. The amplitude of the slip coefficient varies with the flow rate, Q. In Figs. S2a-j, for $\eta \lesssim 5$ mPa·s, the slip coefficient decreases with increasing Q. Whereas, for $\eta \gtrsim 5$ mPa·s, the slip coefficient increases with increasing Q. So, on first sight, the slip coefficient seems to depend on the shear stress. However, the amplitude of variation of s as a function of Q decreases from Figs. S2a to S2e and increases from Figs. S2f to S2j. As shown in Fig. 1e, for flow along D_1, at constant flow rate, Q, the local fluid pressure, P, decreases along the channel from M_1 to M_5. Similarly, for flow in the direction of D_2, the local fluid pressure increases along the channel, from M_1 to M_5 (Fig. 1f). Therefore, the amplitude of variation of s as a function of Q increases with the increase in local pressure. Indeed, the amplitude of variation of s as a function of Q for the same sensor M_1 or M_5, is twice as large in the case of high local pressure (Figs. S2a and S2j) when

compared to low local pressure (Figs. S2e and S2f). Moreover, for sensor M_3, which is placed at the center of the channel, the change in the direction does not change the value of the local pressure. In that case, as shown in Figs. S2c and S2h, the change in direction does not change the amplitude of variation of s as a function of Q. These observations indicate that the slip depends on both the local fluid pressure and shear stress.

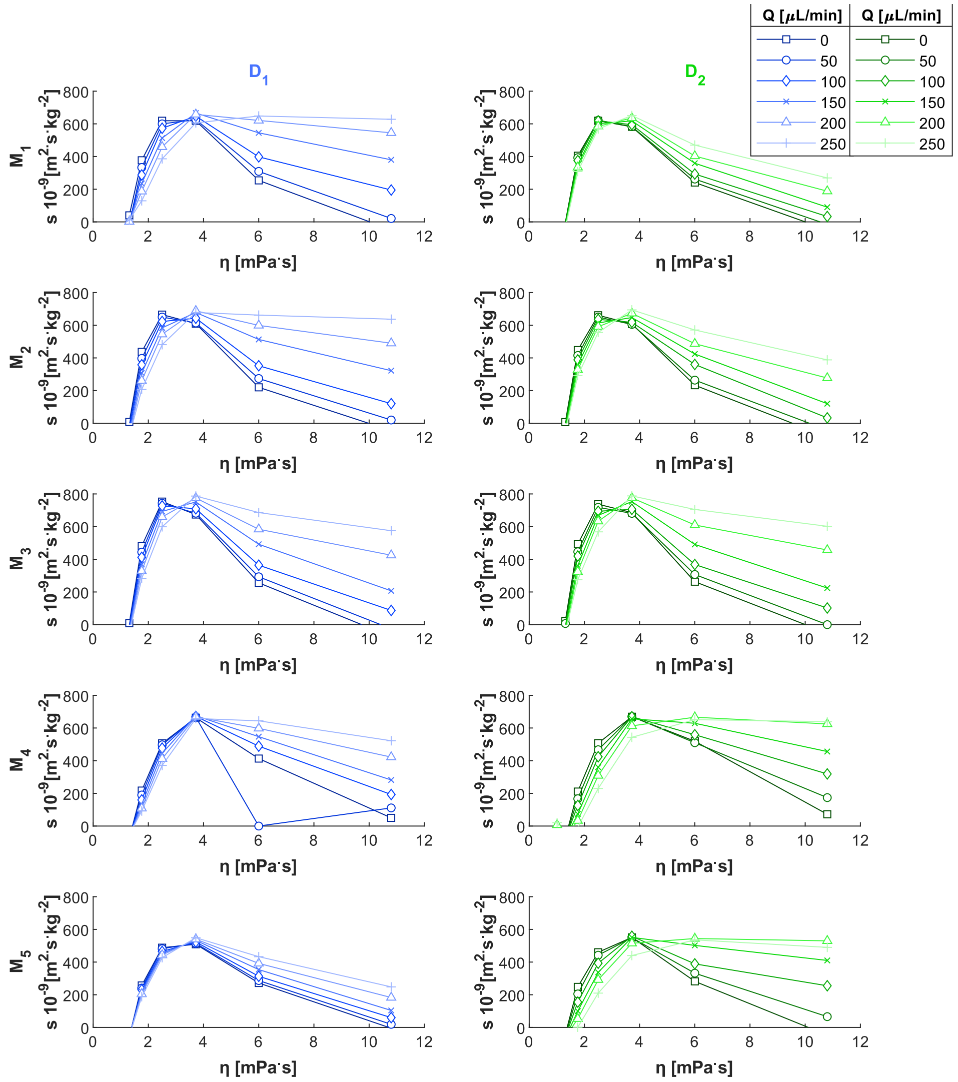

Fig. S2 Effects of pressure and shear stress on the slip coefficient. (a-e) Slip coefficient s for different flow rates, Q, in the direction D_1 as a function of the fluid viscosity, η. The evolution of the slip coefficient along D_1 is represented for three sensors: M_1 in (a), M_2 in (b), M_3 in (c), M_4 in (d) and M_5 in (e). (f-j) Slip coefficient s for different Q, in the direction D_2, as a function of η. The evolution of the slip coefficient along D_2 is represented for three sensors: M_1 in (f), M_2 in (g), M_3 in (h), M_4 in (i) and M_5 in (j). Clearly, the amplitude of s depends on the flow rate Q. Less obvious, the amplitude of s depends also on the local pressure. This result is verifiable with the flow direction comparison of s, for the same sensor and same flow rate.

Statistical information

No flow conditions. Six measurements, n = 6, of the gain, S_{21}, have been performed for each solution of water-glycerol, in case of no flow condition. We calculated, using Eqs. (S4-5), the mean value and the standard error of S_{21} for k = 1 on the linear scale before being expressed in dB. In Fig. 2d-e, the mean value of S_{21}, $< S_{21} >$, and its standard error, ΔS_{21}, are represented by the marker and error bars, respectively.

Mean value of X for n measurements: $$< X > = \frac{\sum_1^n X}{n} \quad \text{(S4)}$$

Standard error of X for n measurements: $$\Delta X = \sqrt{\frac{\sum_1^n (X - <X>)^2}{n(n-1)}} \quad \text{(S5)}$$

Flow conditions. Similarly, three measurements of S_{21} have been performed for each solution, each flow rate, and each direction. For 234 measurements, 78 mean values and uncertainties were extracted. In this table, the ΔS_{21} is lower than 1 % of $< S_{21} >$. Consequently, the uncertainty of S_{21} is smaller than the symbol size in Figs. 3-4, see Table S3. Therefore, in Figs. 3-4, only the mean values of the slip coefficient or of the slip length are presented.

Table S3. Mean value, $< S_{21} >$, and standard error, ΔS_{21}, of the acoustic loss of different water-glycerol solutions, flow rate (Q) and flow direction (D_1 or D_2), for one sensor. Here, for 234 measurements, 78 mean values and uncertainties were extracted. ΔS_{21} is lower than 1 % of $< S_{21} >$.

			$<S_{21}>$ [dB]						ΔS_{21} [dB]						$\Delta S_{21}/<S_{21}>$ [%]					
			w_G						w_G						w_G					
		Q	0	0.1	0.2	0.3	0.4	0.5	0	0.1	0.2	0.3	0.4	0.5	0	0.1	0.2	0.3	0.4	0.5
M_3	D_1	0	-30.81	-32.90	-28.16	-27.05	-27.07	-28.73	0.22	0.00	0.01	0.01	0.02	0.19	0.72	0.02	0.04	0.02	0.06	0.67
		50	-30.46	-32.89	-28.40	-27.26	-26.95	-28.51	0.19	0.00	0.01	0.03	0.00	0.03	0.61	0.01	0.03	0.10	0.02	0.11
		100	-30.24	-32.80	-28.69	-27.46	-26.79	-28.01	0.19	0.01	0.01	0.02	0.01	0.01	0.61	0.03	0.03	0.08	0.04	0.04
		150	-30.03	-32.68	-29.00	-27.72	-26.71	-27.47	0.19	0.01	0.02	0.02	0.00	0.00	0.62	0.04	0.07	0.09	0.02	0.01
		200	-29.84	-32.51	-29.34	-27.99	-26.70	-27.16	0.19	0.00	0.01	0.03	0.01	0.01	0.65	0.01	0.02	0.10	0.04	0.04
		250	-29.66	-32.34	-29.65	-28.31	-26.73	-26.98	0.17	0.02	0.01	0.03	0.02	0.01	0.58	0.06	0.02	0.09	0.08	0.03
	D_2	0	-29.84	-32.95	-28.31	-27.24	-26.99	-28.92	0.01	0.00	0.01	0.03	0.01	0.10	0.04	0.01	0.04	0.13	0.03	0.35
		50	-29.65	-32.93	-28.61	-27.36	-26.83	-28.41	0.01	0.00	0.01	0.00	0.00	0.01	0.03	0.00	0.03	0.02	0.01	0.04
		100	-29.47	-32.85	-28.96	-27.58	-26.73	-27.82	0.00	0.01	0.04	0.00	0.00	0.01	0.02	0.02	0.12	0.01	0.02	0.04
		150	-29.30	-32.72	-29.39	-27.91	-26.72	-27.26	0.01	0.01	0.06	0.05	0.00	0.00	0.02	0.03	0.21	0.18	0.01	0.01
		200	-29.10	-32.57	-29.77	-28.22	-26.80	-27.02	0.00	0.01	0.01	0.04	0.01	0.00	0.01	0.04	0.02	0.15	0.03	0.01
		250	-28.95	-32.40	-30.22	-28.69	-26.92	-26.92	0.01	0.01	0.02	0.05	0.00	0.01	0.03	0.02	0.08	0.19	0.01	0.02

No-slip to slip transition threshold slip coefficient. The threshold slip coefficient is determined by the confidence range of slip. The confidence range is 6-times the standard deviation of s. In Fig. S3, the slip coefficient measurement from the five sensors at six flow rates for both directions D_1 and D_2, and for η=1.31 mPa·s is represented as a function of $\Delta P/\eta$. For this value of viscosity, the fluid does not slip for any value

of $\Delta P/\eta$ and s has a confidence range of ≈ 150 × 10^{-9} $m^2 \cdot s \cdot kg^{-1}$. Therefore, the maximum value of s, which can be used as a no-slip boundary condition, is 150 × 10^{-9} $m^2 \cdot s \cdot kg^{-1}$. This value was used as the threshold to determine the regime transition between no-slip and slip.

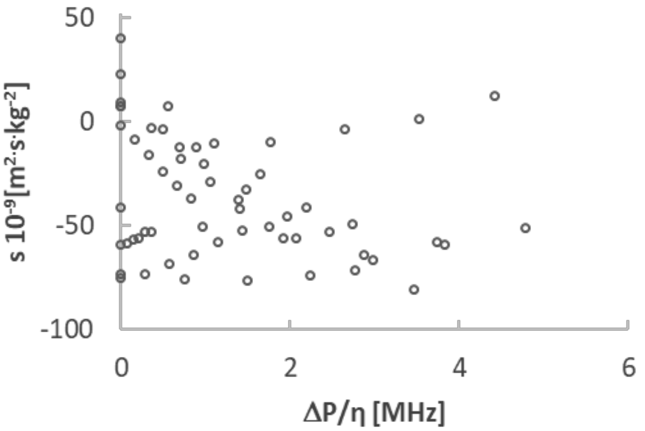

Fig.S3 Confidence interval of slip coefficient. Here we represent the slip coefficient measured with the five sensors, M_1 to M_5, at six flow rates for both flow directions, D_1 and D_2, and for η = 1.31 mPa·s. At this viscosity, the fluid adheres to the crystal, for all flow rates used. The slip coefficient varies from -100 × 10^{-9} $m^2 \cdot s \cdot kg^{-1}$ to 50 × 10^{-9} $m^2 \cdot s \cdot kg^{-1}$. This variation leads to a confidence range of s of 150 × 10^{-9} $m^2 \cdot s \cdot kg^{-1}$.

Supplementary References

[1] Segur, J. B., & Oberstar, H. E. (1951). Viscosity of glycerol and its aqueous solutions. Industrial & Engineering Chemistry, 43(9), 2117–2120. doi:10.1021/ie50501a040

[2] Fergusson, F. A. A., Guptill, E. W., & MacDonald, A. D. (1954). Velocity of sound in glycerol. The Journal of the Acoustical Society of America, 26(1), 67–69. doi:10.1121/1.1907292

[3] Anupama, S., Parameshwara, S., Prashanth, G.R., Renukappa, N. M., & Sundara Rajan, J. (2019). Study of surface energy of SiO2 and TiO2 on charge carrier mobility of rubrene organic field effect transistor. Proceedings of TANN'19, Ottawa, Canada – June, 2019. doi: 10.11159/tann19.135

[4] Zhang, Z., Wang, W., Korpacz, A. N., Dufour, C. R., Weiland, Z. J., Lambert, C. R., & Timko, M. T. (2019). Binary liquid mixture contact-angle measurements for precise estimation of surface free energy. Langmuir. doi:10.1021/acs.langmuir.9b01252

[5] Vicente, C.M.S; André, P.S.; Ferreira, R.A.S. (2012). Simple measurement of surface free energy using a web cam. Revista Brasileira de Ensino de Física, 34(3), –.doi:10.1590/S1806-11172012000300012
